# A comprehensive analysis of infantile central nervous system tumors to improve distinctive criteria for infant‐type hemispheric glioma versus desmoplastic infantile ganglioglioma/astrocytoma

**DOI:** 10.1111/bpa.13182

**Published:** 2023-06-22

**Authors:** Arnault Tauziède‐Espariat, Kévin Beccaria, Volodia Dangouloff‐Ros, Philipp Sievers, Alexandra Meurgey, Daniel Pissaloux, Romain Appay, Raphaël Saffroy, Jacques Grill, Cassandra Mariet, Franck Bourdeaut, Lauren Hasty, Alice Métais, Fabrice Chrétien, Thomas Blauwblomme, Stéphanie Puget, Nathalie Boddaert, Pascale Varlet, the RENOCLIP‐LOC

**Affiliations:** ^1^ Department of Neuropathology, GHU Paris‐Psychiatrie et Neurosciences Sainte‐Anne Hospital Paris France; ^2^ Inserm, UMR 1266, IMA‐Brain Institut de Psychiatrie et Neurosciences de Paris Paris France; ^3^ Department of Pediatric Neurosurgery, Necker Hospital, APHP Université Paris Descartes, Sorbonne Paris Cité Paris France; ^4^ Pediatric Radiology Department Hôpital Necker Enfants Malades, AP‐HP Paris France; ^5^ Université Paris Cité, UMR 1163 Institut Imagine and INSERM U1299 Paris France; ^6^ Department of Neuropathology, Institute of Pathology University Hospital Heidelberg Heidelberg Germany; ^7^ Clinical Cooperation Unit Neuropathology, German Consortium for Translational Cancer Research (DKTK) German Cancer Research Center DKFZ Heidelberg Germany; ^8^ Department of Biopathology Léon Bérard Cancer Center Lyon France; ^9^ INSERM 1052, CNRS 5286 Cancer Research Center of Lyon (CRCL) Lyon France; ^10^ APHM, CHU Timone Service d'Anatomie Pathologique et de Neuropathologie Marseille France; ^11^ Aix‐Marseille University, CNRS, INP, Institute of Neurophysiopathology Marseille France; ^12^ Department of Biochemistry and Oncogenetics Paul Brousse Hospital Villejuif France; ^13^ U981, Molecular Predictors and New Targets in Oncology, INSERM, Gustave Roussy Université Paris‐Saclay Villejuif France; ^14^ Department of Pediatric Oncology, Gustave Roussy Université Paris‐Saclay Villejuif France; ^15^ INSERMU830 Laboratory of Translational Research in Pediatric Oncology Paris France; ^16^ Institut Curie, SIREDO Center Care, Innovation, Research in Pediatric, Adolescent and Young Adult Oncology Paris Sciences Lettres Research University Paris France

**Keywords:** desmoplastic infantile ganglioglioma/astrocytoma, DNA‐methylation, infantile, infant‐type hemispheric glioma

## Abstract

Recent epigenomic analyses have revealed the existence of a new DNA methylation class (MC) of infant‐type hemispheric glioma (IHG). Like desmoplastic infantile ganglioglioma/astrocytoma (DIG/DIA), these tumors mainly affect infants and are supratentorial. While DIG/DIA is characterized by *BRAF* or *RAF1* alterations, IHG has been shown to have receptor tyrosine kinase (RTK) gene fusions (*ALK*, *ROS1*, *NTRK1/2/3*, and *MET*). However, in this rapidly evolving field, a more comprehensive analysis of infantile glial/glioneuronal tumors including clinical, radiological, histopathological, and molecular data is needed. Here, we retrospectively investigated data from 30 infantile glial/glioneuronal tumors, consecutively compiled from our center. They were analyzed by two experienced pediatric neuroradiologists in consensus, without former knowledge of the molecular data. We also performed a comprehensive clinical, and histopathological examination (including molecular evaluation by next‐generation sequencing, RNA sequencing, and fluorescence in situ hybridization [FISH] analyses), as well as DNA methylation profiling for the samples having sufficient material available. The integrative histopathological, genetic, and epigenetic analyses, including t‐distributed stochastic neighbor embedding (t‐SNE) analyses segregated tumors into 10 DIG/DIA (33.3%), six IHG (20.0%), three gangliogliomas (10.0%), two pleomorphic xanthoastrocytomas (6.7%), two pilocytic astrocytomas (6.7%), two supratentorial ependymomas, *ZFTA* fusion‐positive (6.7%), two supratentorial ependymomas, *YAP1* fusion‐positive (6.7%), two embryonal tumors with PLAGL2‐family amplification (6.7%), and one diffuse low‐grade glioma, MAPK‐pathway altered. This study highlights the significant differential features, in terms of histopathology (leptomeningeal infiltration, intense desmoplasia and ganglion cells in DIG/DIA and necrosis, microvascular proliferation, and siderophages in IHG), and radiology between DIG/DIA and IHG. Moreover, these results are consistent with the literature data concerning the molecular dichotomy (*BRAF/RAF1* alterations vs. RTK genes' fusions) between DIG/DIA and IHG. This study characterized histopathologically and radiologically two additional cases of the novel embryonal tumor characterized by *PLAGL2* gene amplification.

## Graphical abstract

Differences between desmoplastic infantile gangliogliomas/astrocytomas and infant‐type hemispheric gliomas.

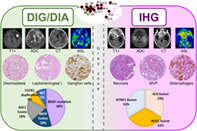

## 1 INTRODUCTION

The diagnosis of infantile brain tumors has recently been revolutionized by molecular biology including genetic and epigenetic data. The last edition of the World Health Organization's (WHO) classification introduced a new infantile histopathological and molecular tumor type, the “infant‐type hemispheric glioma” (IHG) [1]. These tumors are characterized by receptor tyrosine kinase (RTK) alterations (particularly fusions of *NTRK1/2/3*, *ALK*, *ROS1*, or *MET* genes) and a distinct DNA‐methylation profile [1]. They present similarities to desmoplastic infantile ganglioglioma/astrocytoma (DIG/DIA) [2], the well‐known grade 1 benign glial/glioneuronal tumors sharing a common age of onset and a hemispheric location. It was recently discovered that these tumors are driven by the activation of the mitogen‐activated protein kinase (MAPK) pathway, most commonly caused by mutations or fusions involving *BRAF* or *RAF1* [3, 4, 5, 6, 7]. Their classical histopathological features include a prominent desmoplastic leptomeningeal stroma combined with a benign astrocytic differentiation in DIA and a glial and ganglionic differentiation in DIG [1]. A subset of DIG/DIA presenting frank anaplasia or malignant transformation was reported in the pre‐molecular era; however, the literature data are difficult to interpret due to the lack of molecular data [1, 8, 9]. To date, only one study has evidenced a case of DIG/DIA having histopathological signs of malignancy as being clustered within the methylation class (MC) of DIG [10]. Indeed, a subset of molecularly proven IHG has been reclassified from DIG/DIA cases, most likely explaining why some DIG/DIA have been previously described as having RTK fusions or histopathological signs of malignancy [3, 7]. However, Clarke et al. analyzed the DNA‐methylation profiles (using the v11b4 of the DKFZ classifier) of 241 CNS tumors from patients under 4 years of age and demonstrated that the epigenetic distinction between these two infantile tumor types is not always clear (most specimens presented intermediate scores for one of the two MC) [11]. Because IHG has only recently been identified (79 cases reported using DNA‐methylation data), very few data are detailed, particularly in comparison to their main differential diagnosis (DIG/DIA) [10, 11]. Information is limited concerning clinical data (42/79 cases with only survival data, and only one case with detailed treatments), radiology (2/79 cases with some radiological data), histopathology (i.e., the circumscribed vs. diffuse pattern is confusing in the last WHO classification between the DIG/DIA and IHG chapters; no detailed description in 79 cases), immunohistochemistry (no detailed immunoprofile for the 79 reported cases) and genetics (51/79 cases with molecular abnormality). More recently, RTK and MAPK fusions were reported in pediatric cases (including infants) with a DNA‐methylation proven MC of pleomorphic xanthoastrocytomas (PXA) (*ALK*, *ROS1*, *NTRK2*, and *NTRK3* fusions) as well as in the novel MC of glioneuronal tumors, not otherwise specified (NOS), subtype A (*ALK*, *MET*, *NTRK1*, *NTRK2*, *NTRK3*, or *RAF1* fusions) [11, 12, 13, 14, 15, 16]. These data highlight the fact that even with a definite age of diagnosis and location, molecular alterations could be shared by distinct newly recognized histopathological and molecular tumor types, underscoring the importance of an integrated diagnosis. Because all these molecular discoveries are recent, literature data concerning clinical history and radiological characterization are out of date and need to be redefined in light of the integrated diagnoses. Considering the lack of clarity between the different tumor types and the absence of DNA‐methylation profiling for many cases reported in the literature, a diagnostic algorithm has yet to be drawn, particularly for neuropathologists. Herein, we performed an integrative analysis for a cohort of 30 infantile glial/glioneuronal tumors in order to evaluate their genetic landscapes and identify any clinical, radiological, histopathological, or epigenetic (using the v12.5 of the DKFZ classifier) features that might be distinguishing factors for the principal differential diagnoses encountered within this age group.

## 2 METHODS

### 2.1 Study design, patients, and data collection

This study included patients under the age of 12 months diagnosed between 1 January, 1996, and 30 September, 2022 as having a glial/glioneuronal tumor. Initial diagnoses were DIG (*n* = 8), DIA (*n* = 2), anaplastic ependymoma (*n* = 4), glioblastoma (*n* = 4), high‐grade glioma (*n* = 4), low‐grade glioma (*n* = 2), pilomyxoid astrocytoma (*n* = 1), anaplastic pilocytic astrocytoma (*n* = 1), PXA with anaplasia (*n* = 1), dysembryoplastic neuroepithelial tumor (*n* = 1), low‐grade neuroepithelial tumor (*n* = 1), and malignant glioneuronal tumor (*n* = 1). Epidemiological data (gender and age at diagnosis) and data concerning the tumor and treatment (location and size of tumor, extent of resection, relapses, and complementary treatments) were retrospectively analyzed. The extent of the initial resection was assessed by magnetic resonance imaging (MRI) or computed tomography performed after surgery. Informed consent forms were signed by the patients' parents or legal guardians before treatment began. Study approval was obtained by our institutional review board.

### 2.2 Central radiological review

The central radiological review was performed by two experienced pediatric neuroradiologists (NB and VDR). Preoperative MRIs were examined and the following features were analyzed: location, tumor size, signals for T1‐weighted and T2‐weighted sequences, susceptibility imaging, the diffusion and apparent diffusion coefficient (ADC) map, enhancement, presence of cysts, necrosis, and perfusion parameters using arterial spin labeling, when available.

### 2.3 Central pathological review

The central pathology review was performed conjointly by two neuropathologists (ATE and PV). Samples were stained with hematoxylin–phloxin–saffron (HPS) according to standard protocol. For each case, the following pathological features were researched: diffuse or circumscribed growth pattern (based on histopathology—entrapped neurons in the tumor—and using neurofilament staining), microvascular proliferation, tumoral necrosis, calcification, dysmorphic ganglion cells, perivascular mononuclear inflammatory infiltrates, eosinophilic granular bodies, Rosenthal fibers, pseudorosettes, xanthomatous cells, nuclear pseudoinclusions, desmoplasia (on reticulin staining), multinucleated cells, spindle cell components, neuropil islands, embryonal components, and oligodendroglioma‐like components. Mitotic count was monitored using 10 high‐power fields (HPF) which corresponded to 3.2 mm^2^ on our microscope and was counted jointly by two neuropathologists in a hot‐spot area. Integrated diagnoses were performed in accordance with the current WHO classification.

### 2.4 Immunohistochemistry

Unstained 3‐μm‐thick slides of formalin‐fixed paraffin‐embedded (FFPE) tissues were obtained and submitted for immunostaining with an automated stainer (Dako Omnis, Glostrup, Denmark). The following primary antibodies were used: Glial Fibrillary Acidic Protein (GFAP) (1:200, clone 6F2, Dako, Glostrup, Denmark), Olig2 (1:500, clone OLIG2, Sigma‐Aldrich, Saint‐Louis, USA), SOX10 (1:50, clone A‐2, Diagomics, Blagnac, France), neurofilament (1:100, clone NF70, Dako, Glostrup, Denmark), NeuN (1:1000, clone A60, Sigma‐Aldrich, Saint‐Louis, USA), synaptophysin (1:150, clone Synap, Dako, Glostrup, Denmark), chromogranin A (1:200, clone LK2 H10, Diagnostic Biosystem, Pleasanton, USA), CD34 (1:40, clone QBEnd‐10, Dako, Glostrup, Denmark), BRAFV600E (1:100, clone VE1, Abcam, Cambridge, United Kingdom), IDH1R132H (1:40, clone H09, Clinisciences, Nanterre, France), ATRX (1:100, clone BSB‐108, Diagomics, Blagnac, France), EGFR (1:200, clone EGFR.113, Leica, Saint‐Gall, Swiss), PTEN (1:150, clone 6H2.1, Dako, Glostrup, Denmark), H3K27me3 (1:2500, polyclonal, Diagenode, Liege, Belgium), p53 (1:5000, clone DO1, Clinisciences, Nanterre, France), ALK (1:400, clone 1A4, Diagomics, Blagnac, France), ROS1 (1:50, clone EP282, Diagomics, Blagnac, France), Pan‐NTRK (1:100, clone EP1058Y, Abcam, Cambridge, United Kingdom), MSH2 (pre‐diluted, clone FE11, Dako, Glostrup, Denmark), MSH6 (pre‐diluted, clone 44, Dako, Glostrup, Denmark), MLH1 (pre‐diluted, clone E505, Dako, Glostrup, Denmark), PMS2 (pre‐diluted, clone EPR3947, Dako, Glostrup, Denmark), EMA (1:200, clone GM008, Dako, Glostrup, Denmark), NFκB (1:6000, clone D14E12, Cell Signaling Technology, Danvers, USA), L1CAM (1:500, clone UJ127.11, Sigma‐Aldrich, Saint‐Louis, USA), LIN28A (1:150, polyclonal, Ozyme, Saint‐Cyr‐l'Ecole, France), INI1 (1:50, clone 25/BAF47, BD‐Biosciences, Erembodegem, Belgium), and Ki‐67 (1:200, clone MIB‐1, Dako, Glostrup, Denmark). Reticulin staining was performed using the Reticulin silver plating kit according to Gordon & Sweets (Merck Millipore, Guyancourt, France). External positive and negative controls were used for all antibodies and staining. CD34 immunopositivity was considered if there was an extravascular staining. The MIB‐1 labeling index was estimated jointly by two neuropathologists in a hot‐spot area.

### 2.5 Fluorescence in situ hybridization analyses

A fluorescence in situ hybridization (FISH) study was performed on interphase nuclei according to the standard procedures and the manufacturer's instructions. The following probes were used: Vysis CDKN2A/CEP9 FISH Probe Kit (Abbott Molecular, USA), ZytoLight SPEC BRAF dual color break apart probe (Zytovision, Bremerhaven, Germany), ZytoLight SPEC ALK dual color break apart probe (Zytovision, Bremerhaven, Germany), ZytoLight SPEC ROS1 dual color break apart probe (Zytovision, Bremerhaven, Germany), ZytoLight SPEC NTRK1 dual color break apart probe (Zytovision, Bremerhaven, Germany), and ZytoLight SPEC NTRK2 dual color break apart probe (Zytovision, Bremerhaven, Germany). Fluorescent signals were counted in 100 tumoral nuclei with the DM600 Leica fluorescent microscope (Leica Biosystems, Richmond, IL). Deletion and rearrangement were considered if detected in more than 30% and 10% of nuclei, respectively. Results were recorded using a DM600 imaging fluorescence4 microscope (Leica Biosystems, Richmond, IL) fitted with appropriate filters, a CCD camera, and digital imaging software from Leica (Cytovision, v7.4).

### 2.6 Targeted array SNP genotyping

DNA was extracted using the QIAampDNA mini‐kit® (Qiagen Inc., Courtaboeuf, France) according to the manufacturer's protocols. Tissues were disrupted in lysis buffer. After removing the paraffin, the DNA was purified via sequential centrifugation through membrane spin columns. The purity and quantity of DNA were assessed by measuring the absorbance ratio at 260/280 nm with a NanoDrop® Spectrophotometer (LabTech, Palaiseau, France). A brain tumor gene mutation panel was developed using the MassARRAY iPlex technology and MassARRAY online design tools (Agena Bioscience), and included *IDH1* mutations, *IDH2* mutations, *H3F3A* mutations (codon 27–34), *HIST1H3B* mutations (codon 27) *FGFR1* mutations, *TERT* mutations, and *BRAF* mutations (V600E). The MassARRAY iPlex procedure involves a three‐step process consisting of the initial polymerase chain reaction (PCR), inactivation of unincorporated nucleotides by shrimp alkaline phosphatase, and a single‐base primer extension. Afterwards, the products are nano‐dispensed onto a matrix‐loaded silicon chip (SpectroChipII, Ageno Bioscience). Finally, the mutations are detected by matrix‐assisted laser desorption‐ionization–time of flight (MALDI–TOF) mass spectrometry. Data analysis was performed using MassARRAY Typer Analyzer software 4.0.4.20 (Ageno Bioscience), which facilitates the visualization of data patterns as well as the raw spectra.

### 2.7 RNA sequencing

RNA was isolated from FFPE tissues with sufficient tumoral density. It was extracted using the High Pure FFPET RNA Isolation Kit (catalogue # 06650775001, Roche Diagnostics GmbH) in accordance with the manufacturer's instructions. The RNA concentrations were measured on a Qubit 4 Fluorometer (# Q33238, Thermo Fisher Scientific) with the Invitrogen Qubit RNA BR Kit (# Q10210, Thermo Fisher Scientific). The percentage of RNA fragments >200 nt (fragment distribution value; DV200) was evaluated by capillary electrophoresis (Agilent 2100 Bioanalyzer). DV200 >30% was required to process the next steps in the analysis. NGS‐based RNA sequencing was performed using the Illumina TruSight RNA Fusion Panel on a Nextseq550 instrument according to the manufacturer's instructions (Illumina, San Diego, CA, USA). This targeted RNA sequencing panel covers 507 fusion‐associated genes, to assess the most recognized cancer‐related fusions. The TruSight RNA fusion panel gene list is available at https://www.illumina.com/content/dam/illumina‐marketing/documents/products/gene_lists/gene_list_trusight_rna_fusion_panel.xlsx. Seven thousand six hundred and ninety exonic regions are targeted with 21,283 probes. Libraries were prepared according to the Illumina instructions for the TruSight RNA fusion Panel kit. STAR_v2.78a and Bowtie software were used to produce aligned readings in relation to the Homo Sapiens Reference Genome (UCSC hg19). Manta v1.4.0, Tophat2, and Arriba v2.1.0 tools were used for fusion calling. Four cases benefited from a whole‐exome RNA sequencing (WERS) at the Centre Léon Bérard in Lyon, as previously described [17].

### 2.8 DNA methylation profiling

Up to 500 ng of DNA were extracted from each tissue sample (FFPE). All patient samples were analyzed using either Illumina Infinium Methylation EPIC or HumanMethylation450 BeadChip arrays according to the manufacturer's instructions. Affiliation predictions were obtained from a DNA methylation‐based classification web platform for central nervous system tumors (www.molecularneuropathology.org, version v12.5). Data from the EPIC and the 450k methylation array were analyzed using R language (v4.0.4). The minfi package was used to load the idat file and was preprocessed using the preprocess.illumina function with dye bias correction and background correction. We removed probes located on sex chromosomes, those not uniquely mapped to the human reference genome (hg19), probes containing single nucleotide polymorphisms, and probes not present in the EPIC or 450 k methylation array. A batch effect correction was effectuated by the removebatchEffect function from the limma package, to remove differences between the FFPE and frozen samples. The probes were sorted by standard deviation with the 10,000 most variable probes being kept for the clustering analysis. These probes were used to calculate the 1‐variance weighted Pearson correlation between samples. The distance matrix was used as input for t‐distributed stochastic neighbor embedding (t‐SNE) from the Rtsne package, using the non‐default parameters: theta = 0, pca = F, max_iter = 2500, and perplexity = 20. Visualization was achieved using ggplot2 packages.

### 2.9 Statistics

Quantitative variables are expressed by median and compared using Mann–Whitney tests. Qualitative variables are expressed by proportions and percentages and compared using Fischer's exact test.

Progression‐free survival, termed PFS, was defined as the time between the first treatment and the first sign of radiologically confirmed progression by MRI. Overall survival (OS) was calculated as the time from the date of diagnosis to the date of death from any cause or the date of the last follow‐up. Patients were considered disease‐free (DF) if gross total resection was achieved and no sign of disease recurrence was detected at the last follow‐up. The appearance of new leptomeningeal contrast enhancement and/or distant metastases, and/or growth at the main tumor site, as assessed by MRI, were considered disease progression (DP).

Censored variables were analyzed using the Kaplan–Meier method and comparisons were assessed using the log‐rank test. These analyses were performed using GraphPad Prism version 9.4.1. All statistical significance was considered at a 5% alpha level.

## 3 RESULTS

### 3.1 The integrated diagnosis identified eight distinct tumor types

**FIGURE 1:**
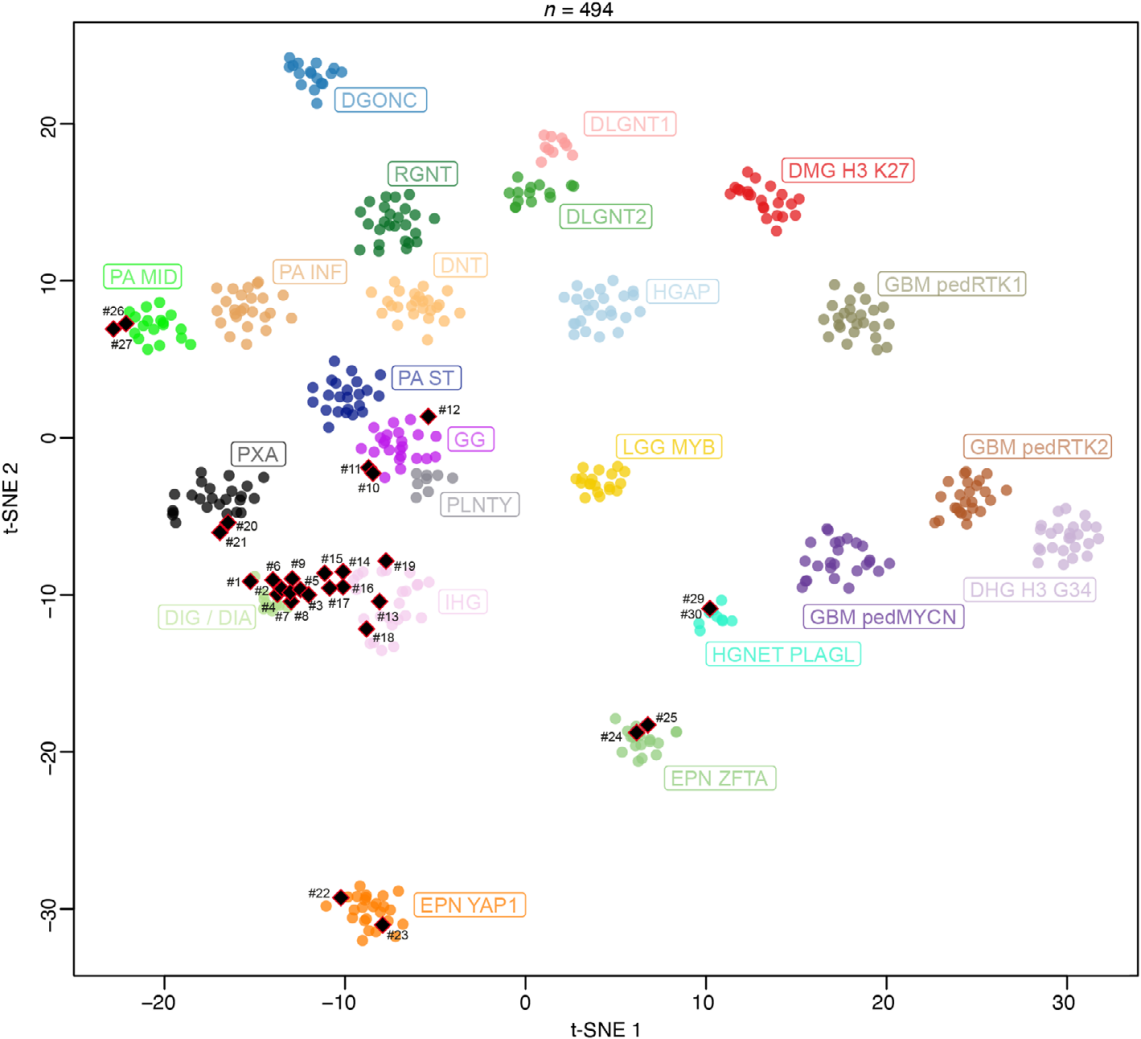
DNA methylation‐based t‐distributed stochastic neighbor embedding distribution. Our tumors were compared to the following reference DNA methylation classes. Diffuse glioneuronal tumor with oligodendroglioma‐like features and nuclear clusters (DGONC), diffuse hemispheric glioma, H3.3 G34 mutant (DHG H3 G34), desmoplastic infantile ganglioglioma/desmoplastic infantile astrocytoma (DIG/DIA), diffuse leptomeningeal glioneuronal tumor, subtype 1 (DLGNT1), diffuse leptomeningeal glioneuronal tumor, subtype 2 (DLGNT2), diffuse midline glioma H3 K27M mutant (DMG H3K27), dysembryoplastic neuroepithelial tumor (DNT), supratentorial ependymoma, YAP1 fusion‐positive (EPN YAP1), supratentorial ependymoma, ZFTA fusion‐positive (EPN ZFTA), pediatric glioblastoma, IDH wildtype, subclass MYCN (GBM pedMYCN); pediatric glioblastoma, IDH wildtype, subclass RTK1 (GBM pedRTK1), pediatric glioblastoma, IDH wildtype, subclass RTK2 (GBM pedRTK2), ganglioglioma (GG), high‐grade astrocytoma with piloid features (HGAP), high‐grade neuroepithelial tumor, with PLAG‐family amplification (HGNET PLAGL), infant‐type hemispheric glioma (IHG), low‐grade glioma, MYB (LGG MYB), infratentorial pilocytic astrocytoma (PA INF), midline pilocytic astrocytoma (PA MID), supratentorial pilocytic astrocytoma (PA ST), polymorphous low‐grade neuroepithelial tumor of the young (PLNTY), pleomorphic xanthoastrocytoma (PXA), and rosette forming glioneuronal tumor (RGNT).

**FIGURE 2:**
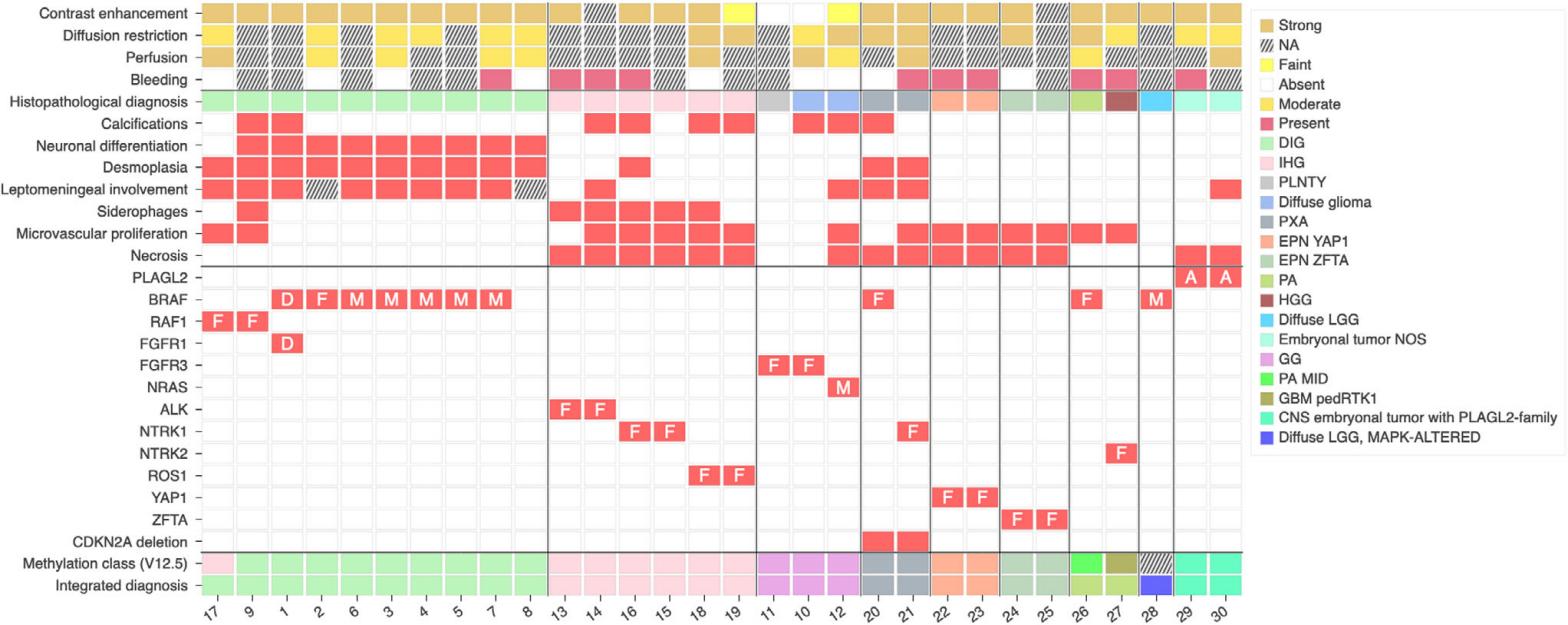
Radiological, histopathological characteristics and recurrent genetic alterations of the 30 tumors. A, amplification; CNS, central nervous system; D, duplication; DIG, desmoplastic infantile ganglioglioma; EPN, ependymoma; F, fusion; GBM, glioblastoma; GG, ganglioglioma; HGG, high‐grade glioma; IHG, infant‐type hemispheric glioma; LGG, low‐grade glioma; M, mutation; MID, midline; NA, not available; NOS, not otherwise specified; PA, pilocytic astrocytoma; ped, pediatric; PLNTY, polymorphous low‐grade neuroepithelial tumor of the young; PXA, pleomorphic xanthoastrocytoma.

Based on histopathological, genetic, and epigenetic analyses, tumors were diagnosed as: 10 DIG (33.3%), six IHG (20%), three gangliogliomas (10%), two PXA (6.7%), two supratentorial ependymomas, *ZFTA‐*fusion positive (6.7%), two supratentorial ependymomas, *YAP1‐*fusion positive (6.7%), two pilocytic astrocytomas (PA) (6.7%), two CNS embryonal tumors, with *PLAGL2* amplification (6.7%), and one diffuse low‐grade glioma (DLGG), MAPK pathway‐altered (*BRAF‐*mutant, 3.3%), (Figures 1 and 2). Using DNA‐methylation profiling, we performed a t‐SNE analysis of the whole cohort to better classify tumors with low calibrated scores (<0.9) (Figure 1).

**FIGURE 3:**
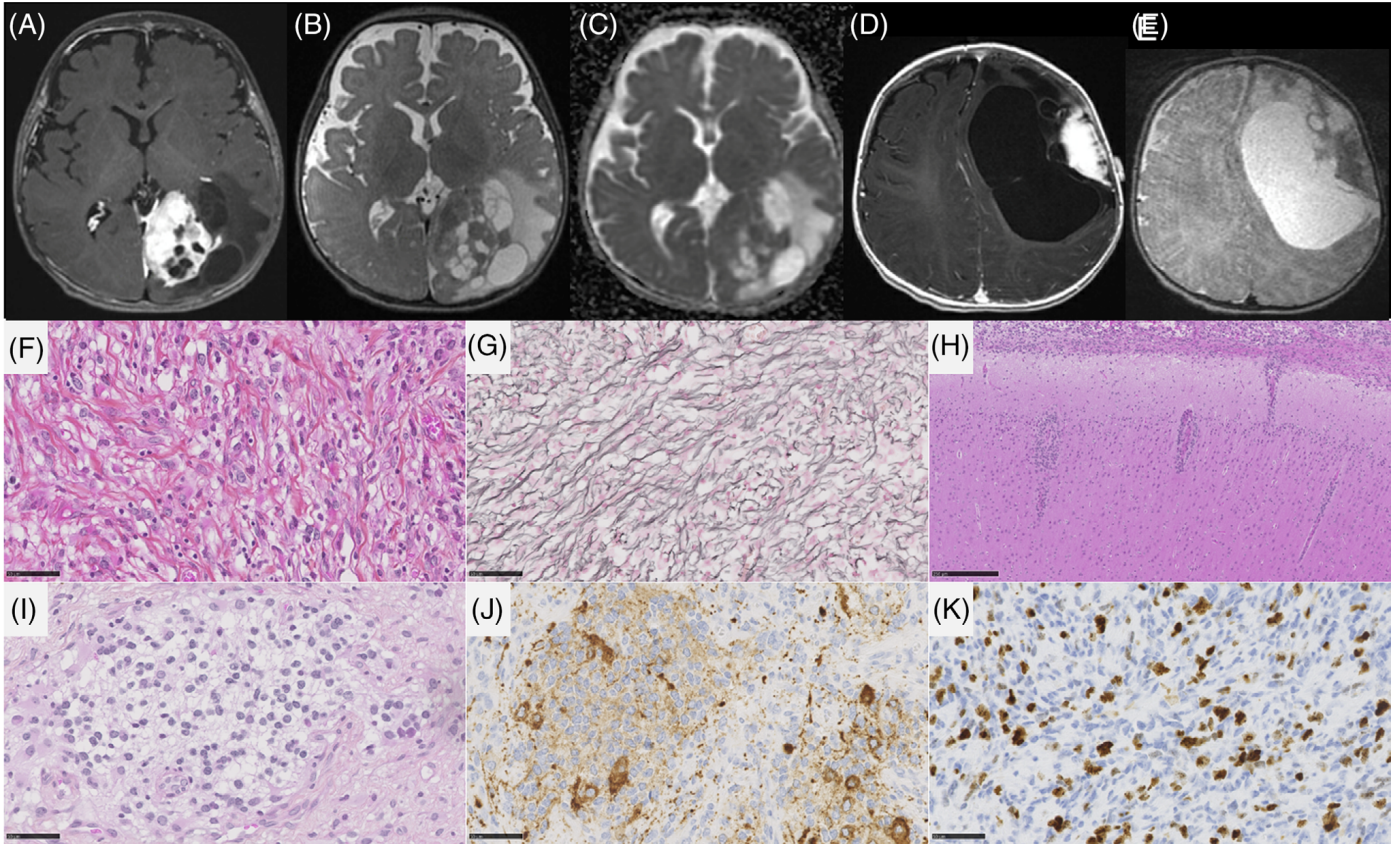
Imaging and histopathological features of desmoplastic infantile gangliogliomas/astrocytomas. MR images of case #7: (A) a left occipital mass with dural contact. The tissular part display avid contrast enhancement, while cysts walls did not enhance. (B) The mass has a median tissular part and large peripheral cysts on T2‐weighted images. (C) ADC map shows intermediate diffusion restriction in the tumoral tissue. (D) MR images of case #4, showing a left frontal mass with dural contact. The tissular part display avid contrast enhancement, while cysts walls did not enhance. (E) The mass has a lateral tissular part and large cysts on T2‐weighted images. (F) A glial proliferation with a collagen matrix (case #4, HPS, magnification 400×), highlighted by the reticulin staining (case #4, G, magnification 400×). (H) Infiltration of the leptomeninges and the Virchow‐Robin spaces (case #4, HPS, magnification 400×). (I) Neuronal differentiation with neurocytic cells (case #4, HPS, magnification 400×) and expression of synaptophysin (case #4, J, magnification 400×). (K) Elevated MIB1 labeling index (case #3, magnification 400×). Black scale bars represent 50 μm. ADC, diffusion coefficient map; HPS, hematoxylin–phloxin–saffron; MR, magnetic resonance.

DIG presented a biphasic morphology with a predominant desmoplastic leptomeningeal component admixed with a neuroepithelial component containing astrocytic and neuronal cells (Figure 3 and Table S1 for details). For all cases, reticulin staining confirmed the presence of a collagen matrix intermixed with spindle cells. A leptomeningeal infiltration was detected in all cases, and two cases presented a tumoral invasion of the Virchow‐Robin spaces in the adjacent brain parenchyma. Ganglion cells and neurocytic differentiation, confirmed by the expression of neuronal markers (synaptophysin, chromogranin A, and NeuN), were observed in all cases, and eosinophilic granular bodies were seen in 3/10 cases. Six out of 10 cases contained foci of embryonal‐like tumor cells. All tumors were well‐circumscribed from the brain parenchyma using neurofilament staining. Calcifications were present in 3/10 cases. No perivascular mononuclear inflammatory infiltrates, Rosenthal fibers, or xanthomatous cells were observed. Necrosis was absent and microvascular proliferation was only focally observed in 2/10 cases. Mitotic count (median mitotic count = 2, varying from 0 to 36), and proliferative index were highly variable (low in desmoplastic and astrocytic and neuronal components and high in embryonal‐like tumor cells). CD34 extravascular positivity was observed in the tumor cells of 2/10 cases. Alterations of *BRAF* (7/10, including 5 mutations, 1 fusion, and 1 duplication) and *RAF1* (2/10, only fusions) were present. No alteration of the MAPK pathway was found in the latter case. Finally, no *CDKN2A* deletion was present. From the 10 DIG classified by histopathology and genetic analyses, eight were confirmed using DNA‐methylation profiling with high calibrated scores (>0.9). One case (#9) classified as DIG/DIA (with a calibrated score of 0.50) definitively clustered within this MC by t‐SNE analysis. The remaining case (#17) was classified as an IHG (score >0.9). However, it harbored a *PRKAR2A::RAF1* fusion, and presented two components: a dominant desmoplastic component and a more cellular and highly proliferative component with numerous mitoses and an elevated proliferative index. Using t‐SNE analysis, the classification of this case was less clear than the predicted score and clustered between IHG and DIG/DIA. After integrating all histopathological and genetic data, the final diagnosis was a DIG, with *RAF1* fusion.

**FIGURE 4:**
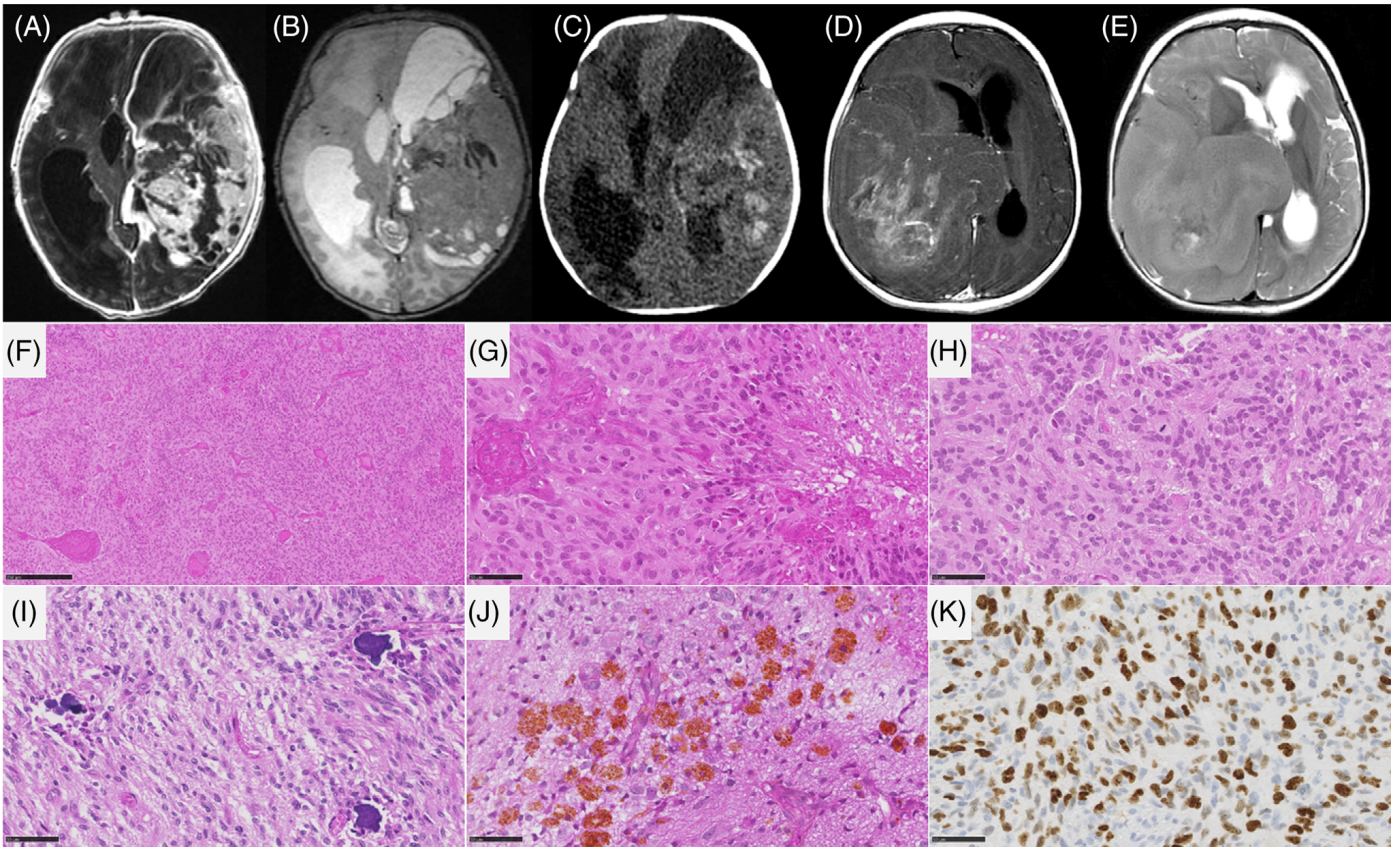
Imaging and histopathological features of infant‐type hemispheric gliomas. (A–C) MR images of case #6, showing a large mass centered in the left temporal lobe, with dural contact. The mass has tissular and cystic parts on T2‐weighted images (B). The tissular part and cysts walls display avid contrast enhancement (A). CT scanner (C) shows tumoral bleeding (signs of an old hemorrhage). (D, E) MR images of case #19, showing a right temporo‐parietal mass with dural contact. The mass has mostly tissular content on T2‐weighted images, with central necrosis (E). The tumor display heterogeneous incomplete enhancement after gadolinium injection (D). (F, G) Densely cellular glial proliferation with necrosis and microvascular proliferation (case #15, HPS, magnification 100× and 400×). (H) Numerous mitoses (case #15, HPS, magnification 400×). (I, J) Presence of calcifications and siderophages (case #16, HPS, magnification 400×). (K) Elevated MIB1 labeling index (case #15, magnification 400×). Black scale bars represent 250 μm (F), and 50 μm (G–K). CT, computerized tomodensitometry; HPS, hematoxylin–phloxin–saffron; MR, magnetic resonance.

IHG were densely cellular and well‐circumscribed (5/6 cases) from the adjacent brain parenchyma (Figure 4). A desmoplastic leptomeningeal component was only observed in 1/6 cases and was focal. Invasion of the Virchow‐Robin spaces was also only present in one case. Tumor cells presented either ependymal (4/6 cases), astrocytic (gemistocytic pattern in 2/6 cases), or oligodendroglioma‐like differentiation (4/6 cases). All cases but one presented necrosis (palisading necrosis in 5 cases), an overall high mitotic count (median = 29, varying from 6 to 41), and prominent glomeruloid microvascular proliferation (5/6 cases). Five cases presented hemorrhagic modifications with siderophages (deposition of iron pigment in macrophages suggesting a past hemorrhage). Calcifications were present in 4/6 cases. No ganglion cells, perivascular inflammatory infiltrates, eosinophilic granular bodies, or xanthomatous cells were observed and neuronal markers were only focally expressed in 1/6 cases. No CD34 immunoreactivity was observed. Genetic alterations included fusions involving: *ALK* (2/6 cases), *NTRK1* (2/6 cases), and *ROS1* (2/6 cases). No *CDKN2A* deletion was present. Five cases were confirmed using DNA‐methylation profiling with high calibrated scores (>0.9). The last case (#15) classified as IHG (with a calibrated score of 0.27) was clustered within this MC using t‐SNE analysis.

Two cases were classified histopathologically and using DNA‐methylation profiling (score >0.9) as PXA. They presented classical features (pleomorphic tumor cells, spindle cells, xanthomatous cells, eosinophilic granular bodies with reticulin staining surrounding individual cells, and CD34 immunopositivity), and a homozygous deletion of *CDKN2A*. Genetic alterations include one *CLIP2::BRAF* fusion and one *TPM3::NTRK1* fusion. Both presented brisk mitotic activity and palisading necrosis (one of them presented a focal glomeruloid microvascular proliferation).

Ependymomas, *ZFTA‐*fusion positive and ependymomas, *YAP1‐*fusion positive presented classical features of ependymoma with a nuclear accumulation of NFκB nuclear protein for the first group and eosinophilic granular bodies and strong immunoreactivity for EMA, without nuclear expression of NFκB for the second group. The DNA‐methylation profiling of these four cases was in line with those diagnoses (score >0.9).

**FIGURE 5:**
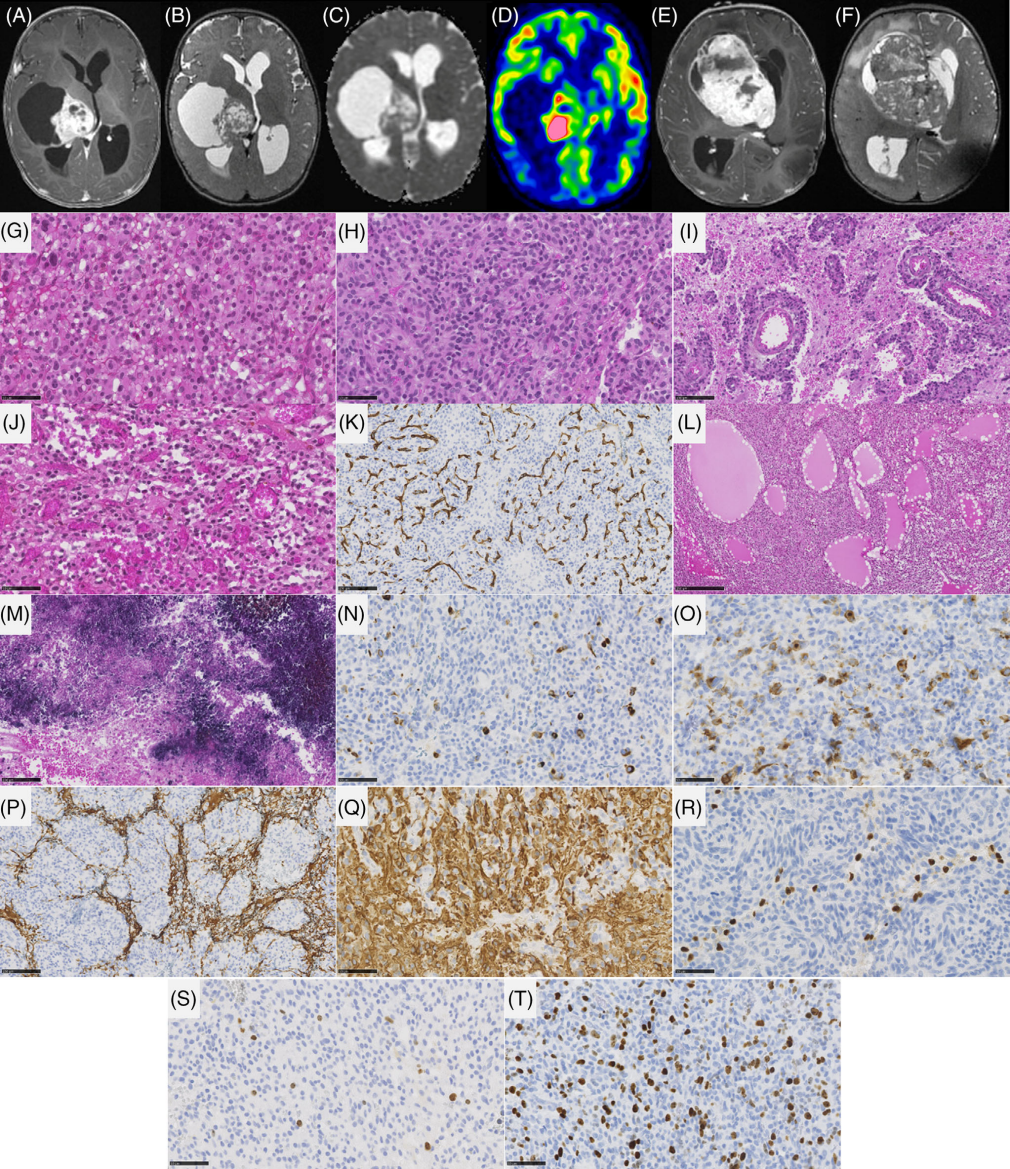
Imaging and histopathological features of embryonal tumors, with PLAGL2 amplification. (A–D) MR images of case #30, showing a right thalamic mass. The mass has cystic and tissular content on T2‐weighted images (B). The tissular part display avid contrast enhancement (A), intermediate diffusion restriction (C), very high cerebral blood flow using ASL (157 mL/min/100 g) (D). (E, F) MR images of case #29, showing a right caudate nucleus mass. The mass has tissular (mostly) and cystic content on T2‐weighted images (F). The tissular part display avid contrast enhancement (E). (G, H) Densely cellular neoplasm composed of monotonous round or spindle tumor cells (case #30, HPS, magnification 400×). (I) Perivascular disposition of tumor cells (case #30, HPS, magnification 200×). (J, K) Tumor with a dense capillary network without microvascular proliferation (case #30, HPS, magnification 400×, and CD34, magnification 200×). (L) Microcystic modifications (case #30, HPS, magnification 100×). (M) Necrosis with calcifications (case #30, HPS, magnification 200×). (N, O) A subset of tumor cells express neuronal markers (case #30, neurofilament, magnification 400×, and synaptophysin, magnification 400×). (P) Residual expression of GFAP without staining of tumor cells (case #30, magnification 200×). (Q) Strong expression of GFAP by tumor cells in another case (case #29, magnification 400×). (R) No immunoexpression of Olig2 by tumor cells (case #30, magnification 400×). (S, T) Variable MIB labeling index (case #29 and 30, magnification 400×). Black scale bars represent 250 μm (L), 100 μm (I, K, M, P), and 50 μm (G, H, J, N, O, Q–T). ASL, arterial spin labeling; HPS, hematoxylin–phloxin–saffron; MR, magnetic resonance.

Two cases (#29 and 30), histopathologically diagnosed as glioneuronal tumors, NEC, were classified as CNS embryonal tumors with PLAG‐family amplification (Figure 5). They presented a *PLAGL2* gene amplification by CNV (copy number variation) analysis and showed the same histopathological features. They were well‐circumscribed from the brain parenchyma and composed of sheets of monotonous oval cells with round to oval nuclei and a pale cytoplasm. In some areas, an epithelioid pattern was present with sharply demarcated tumor cells. A dense branching capillary network (without microvascular proliferation) was present. No rosettes, pseudorosettes or rhabdoid component were present. Hemorrhagic and microcystic modifications were present. Necrosis (not palisading) was constantly observed. The mitotic count and proliferation index were variable (low in one case and high in the other). Using immunohistochemistry, tumor cells presented a preserved expression of INI1, BRG1, and there was no immunopositivity for LIN28A or BCOR. Glial markers were only focally expressed (focal immunoexpression of GFAP without any reactivity for Olig2 and SOX10) and neuronal markers were constantly expressed.

Two cases were classified as PA. One of them (#26) presented the histopathological features of a pilomyxoid astrocytoma (myxoid modifications, piloid cytology, and an angiocentric pattern, without Rosenthal fibers or eosinophilic granular bodies). Genetic analyses revealed the presence of a *KIAA1549::BRAF* fusion. The second case (#27), harboring a *BCR::NTRK2* fusion, showed a biphasic tumor with an oligodendroglioma‐like and piloid cytology (without Rosenthal fibers or eosinophilic granular bodies) and an angiocentric pattern. The central radiological review (cf. above) confirmed that the tumor infiltrated the optic chiasma. Because of a high mitotic count, the case was initially diagnosed as a high‐grade glioma but the DNA‐methylation profiling, using t‐SNE analysis, reclassified it as a midline PA. The last case (#27), which was classified as a diffuse pediatric‐type HGG, RTK1 subtype (with a calibrated score of 0.66), was in close vicinity to PA, midline by t‐SNE analysis. According to all these data, the integrated diagnosis was PA, with *NTRK2* fusion.

**FIGURE 6:**
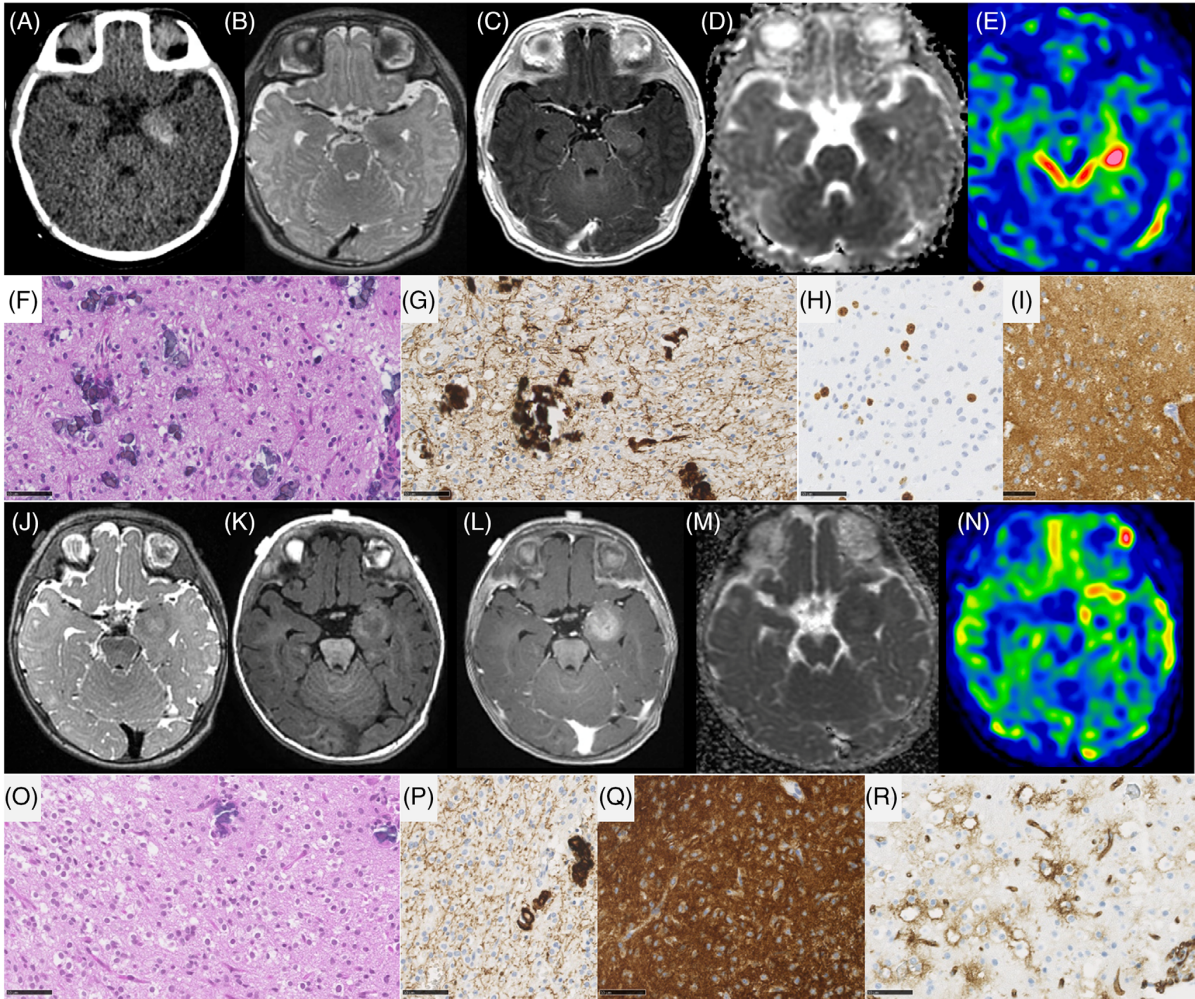
Imaging and histopathological features of gangliogliomas. (A–D) MR images of case #10, showing a left internal temporal mass with tissular content. The mass has microcalcifications on CT (A), low signal on T2‐weighted images (B), faint contrast enhancement (C), restricted diffusion on ADC map (D), and intermediate cerebral blood flow using ASL (75 mL/min/100 g) (E). (F) Diffuse glial proliferation with calcifications (case #10, HPS, magnification 400×). (G) Diffuse growth pattern using neurofilament staining (case #10, magnification 400×). (H) MIB1 labeling index (case #10, magnification 400×). (I–M) MR images of case #12, showing a left internal temporal mass with tissular content. The mass has heterogeneous low signal on T2‐weighted images (I), hyperintense signal on T1‐weighted images pre‐contrast (J), weak contrast enhancement (K), restricted diffusion on ADC map (L), and intermediate cerebral blood flow using ASL (56 mL/min/100 g) (M). (N) Diffuse glial proliferation with calcifications and oligodendroglial‐like features (case #11, HPS, magnification 400×). (O) Diffuse growth pattern using neurofilament staining (case #11, magnification 400×). (P) Diffuse and strong expression of FGFR3 (case #11, magnification 400×). (Q) CD34 extravascular expression (case #11, magnification 400×). (R) Diffuse and strong expression of FGFR3 (case #11, magnification 400×). Black scale bars represent 50 μm. ADC, diffusion coefficient map; ASL, arterial spin labeling; HPS, hematoxylin–phloxin–saffron; MR, magnetic resonance.

Three cases were classified as gangliogliomas using DNA‐methylation profiling (all with calibrated scores >0.9) (Figure 6). Interestingly, none of them presented classical histopathological features of ganglioglioma and two of them had histological signs of aggressiveness (#10 and 12). Among them, case #11 was initially diagnosed as PLNTY because the tumor presented a diffuse growth pattern with an oligodendroglioma‐like component, few mitotic figures, and CD34 extravascular immunopositivity. Genetic analyses revealed the presence of a *FGFR3::TACC3* fusion. One another case (#10) presented the same features as case #11 but without CD34 immunoreactivity. It showed a high mitotic count (6 mitoses/10 HPF) and an elevated proliferative index (12%). There was no microvascular proliferation, no necrosis, and no morphological features suggestive of an alternative tumor type. Genetic analyses revealed the presence of a *FGFR3::TACC3* fusion. The last case (#12) presented as a diffuse glioma with high mitotic activity (5 mitoses/10 HPF) and an elevated proliferative index (15%) associated with necrosis and microvascular proliferation. Genetic analyses revealed a *NRAS* mutation.

The last case (#28) of the cohort presented a diffuse growth pattern with an oligodendroglioma‐like component, and few mitotic figures, but did not show CD34 immunopositivity. Using immunohistochemistry, tumor cells expressed BRAFV600E immunoreactivity. After integrating all these data, the final diagnosis was DLGG, MAPK pathway‐altered.

### 3.2 Distinct histopathological features between IHG and DIG

Ganglionic cells, desmoplasia, and leptomeningeal involvement were significantly and more frequently observed in DIG (respectively *p* = 0.0009, *p* = 0.001, and *p* = 0.003), however siderophages, microvascular proliferation, and necrosis were significantly and more frequently observed in IHG (respectively *p* = 0.007, *p* = 0.035, *p* = 0.0001). The median mitotic count was 2 [1; 16.3] mitoses per 1.6 mm^2^ for DIGs and 29 [7; 37] mitoses per 1.6 mm^2^ for IHG, with no statistically significant difference (*p* = 0.06). No case of either DIG or IHG displayed Rosenthal fibers, rosettes, or xanthomatous cells. Pseudorosettes were present in 50% (3/6) of IHG and in only one DIG (10%, *p* = 0.11). Calcifications were displayed in 4/6 IHG and 2/10 DIG (*p* = 0.12). DIG displayed perivascular inflammatory infiltrates in 50% (5/10) and eosinophilic granular bodies in 30% (3/10) of cases, whereas neither were present in IHG (respectively *p* = 0.09 and *p* = 0.25).

### 3.3 Clinico‐radiological correlation with integrated diagnoses

Relevant clinical data are summarized in Table S1. The median age at diagnosis was 0.6 years (patients' ages ranged from 0 to 1 year). The male/female sex ratio was 0.8 (13 males and 17 females). Symptoms varied according to the location of tumors. Seizures seem to be associated more with the tumor groups of ganglioglioma and DIG/DIA (73% of cases, 8/11) and intracranial hypertension syndrome with IHG.

DIG/DIA, IHG (Figures 3 and 4), and PXA shared several imaging features: large tumors (median volume 124 cm^3^, IQR [49–229]), with leptomeningeal and dural contact, strong contrast enhancement, peritumoral edema. Large tumoral cysts were seen in 90% of DIG/DIA, 50% of IHG, and 100% of PXA. These cysts had enhancing walls in IHG and PXA, as opposed to the non‐enhancing walls of DIG/DIA cysts. Other distinctive features were tumoral bleeding, suggesting a past hemorrhage (0% of DIG/DIA, 75% of IHG, and 50% of PXA), the diffusion restriction (moderate in DIG/DIA, strong in IHG and PXA), and the perfusion data when available (moderate in DIG/DIA (range of maximal cerebral blood flow in 5 patients 40–80 mL/min/100 g). It was found to be very high in one IHG (117 mL/min/100 g) and one PXA (235 mL/min/100 g)). Tumoral locations slightly differ, DIG/DIA being located in every lobe, while IHG and PXA have a temporo‐parietal epicenter. The ependymomas, *YAP1‐*fusion positive had similar imaging features with large tumors, peritumoral edema, and dural contact, but their contrast enhancement was weaker, and the tumors possessed central necrosis without a real cystic component. The ependymomas, *ZFTA‐*fusion positive had similar characteristics but had intra‐parenchymal locations without leptomeningeal contact. The three ependymomas had microcalcifications on CT which were absent in the previous tumor types. The two tumors having *PLAGL2* amplification were large tumors with a cystic component, deeply localized with basal ganglia involvement, intermediate diffusion restriction, and high cerebral blood flow (157 mL/min/100 g) (Figure 5).

PA had different imaging characteristics, as being located within the internal temporal lobe with the involvement of visual pathways. They were large in size, with no cystic component, no peritumoral edema, high contrast enhancement, low perfusion values, tumoral bleeding, and no diffusion restriction.

Finally, gangliogliomas and the DLGG, MAPK pathway‐altered, had strikingly different imaging features from the other tumors (Figure 6). They were much smaller (median volume 3 cm^3^, IQR [2–5], *p* = 0.004), and located in the internal temporal lobe in contact with the Sylvian valley, with tissular content (no cyst), without contrast enhancement, without bleeding, with restricted diffusion, intermediate perfusion values (range 56–75 mL/min/100 g) and without peritumoral edema.

Outcome data were available for 28/30 patients included in the cohort. Twelve (46%) of the patients had tumor recurrence, with a mean PFS of 16 months (median 6.5 months; 95% CI: 1–120). To note, PA and PXA have a tumor recurrence rate of 100%. Four patients with different tumor types (two IHG, one PA, and one PXA), died of their disease, with a mean OS of 16 months. Of these patients, only one had undergone total resection. At the end of follow‐up, no patients with DIG/DIA died of their disease whereas 33.3% (2/6) of patients with a diagnosis of IHG had a fatal outcome.

## 4 DISCUSSION

**FIGURE 7:**
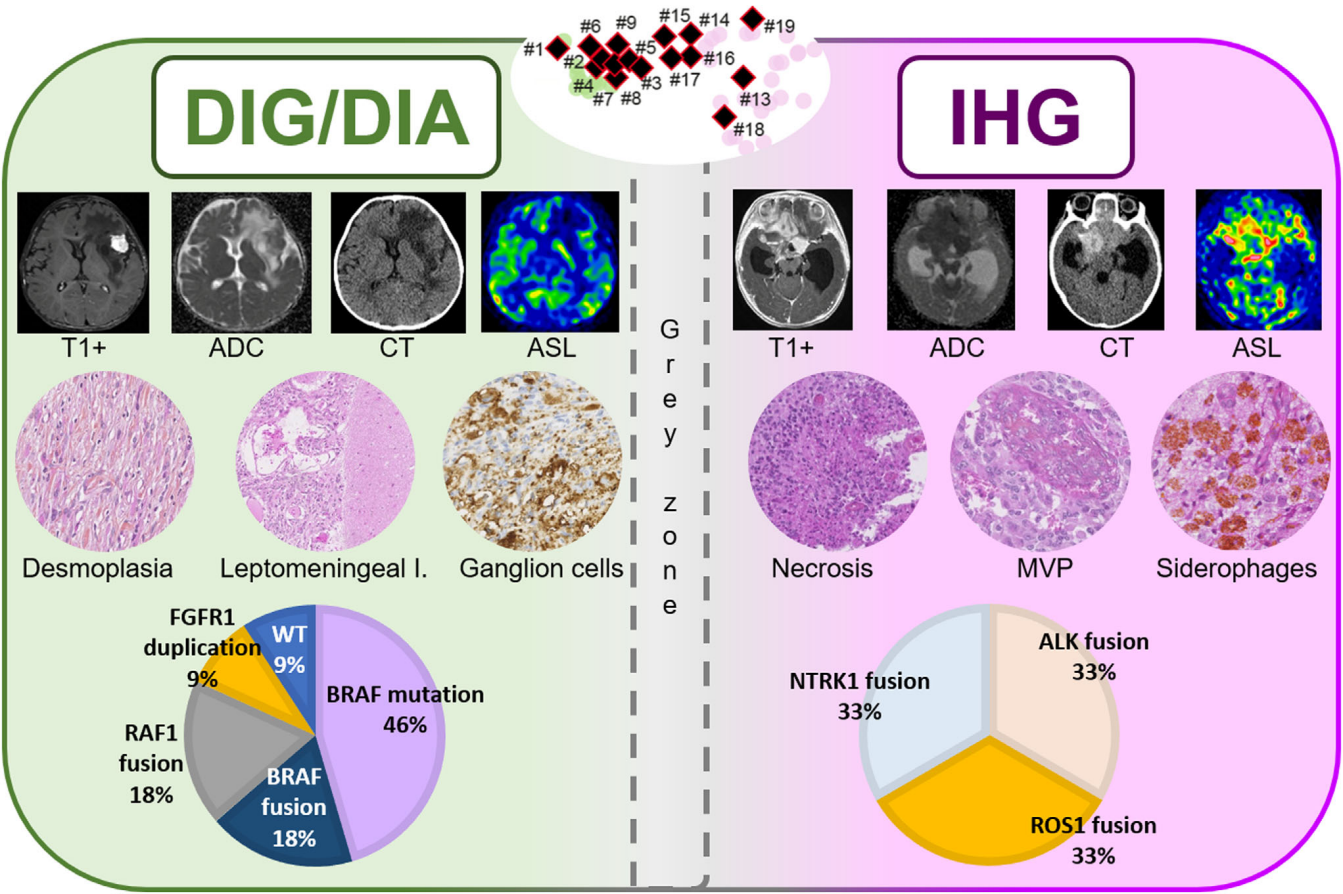
Summary of desmoplastic infantile gangliogliomas/astrocytomas and infant‐type hemispheric gliomas main characteristics. Both DIG/DIA and IHG displayed avid contrast enhancement, but cysts walls of DIG/DIA did not enhance, in comparison with enhancing cysts walls of IHG. Diffusion restriction on ADC maps was moderate in DIAG/DIA patients and strongly restricted in IHG. IHG had frequent bleeding on CT, and not DIA/DIG. Perfusion values using ASL were moderate in DIG/DIA patients and high in IHG. DIG/DIA presents desmoplasia, infiltration of the leptomeninges, and a neuronal differentiation whereas IHG showed obvious signs of malignancy (necrosis and MVP), and siderophages (suggesting a past hemorrhage). Molecular alterations are distinct between DIG/DIA (showing MAPK genes alterations) and IHG (showing RTK gene fusions). DNA‐methylation profiles of DIG/DIA and IHG are distinct but a grey zone exists with overlapping cases. ADC, diffusion coefficient map; ASL, arterial spin labeling; CT, computerized tomodensitometry; DIG/DIA, desmoplastic infantile agangligliomas/astrocytomas; I, involvement; IHG, infant‐type hemispheric gliomas; MAPK, mitogen‐activated protein kinase; MVP, microvascular proliferation; RTK, receptor tyrosine kinase; WT, wildtype.

In line with the literature, this work showed that infantile glial/glioneuronal tumors are mainly represented by DIG/DIA and IHG [2, 11]. This last tumor type was recently identified based on genetic alterations and DNA‐methylation profiling from cohorts of infantile tumors, some of them being initially diagnosed as DIG/DIA [2, 11]. Moreover, Clarke et al. evidenced a potential epigenetic overlap/continuum between IHG and DIG/DIA and that the boundaries between these two MC were not clear, leaving a high proportion of cases remaining unclassified by DNA‐methylation analyses [11]. Because these studies focused on genetic and epigenetic results, very few data are available concerning the differential clinical, radiological, histopathological, and genetic diagnostic features concerning these two entities. In the current series, we showed that DIG/DIA and IHG share some radiological (voluminous circumscribed mass with cystic and solid portions, and dural attachment) and histopathological (mainly well‐circumscribed tumors) features but may be distinguished by several aspects (Figure 7). Firstly, IHG cyst walls seem to present a more intense enhancement after injection of gadolinium than those of DIG/DIA, even if the tissular component is strongly enhanced in both tumor types. Also, while DIG/DIA presents a prominent desmoplastic component, a fibrous stroma is only rare and focal in IHG. Moreover, IHG shows obvious and evenly distributed signs of malignancy (highly cellular tumors, prominent glomeruloid microvascular proliferation, palisading necrosis explaining the intense enhancement by MRI) whereas these signs are focal or limited to the embryonal‐like component of DIG/DIA. This corresponds to the stronger diffusion restriction and higher perfusion values in IHG. The neuronal differentiation (gangliocytic pattern, neuronal cells, and expression of neuronal markers) is frequently observed in DIG/DIA, whereas it is rare and focal in IHG. However, IHG may present an ependymal differentiation which is not observed in DIG/DIA. As previously described, genetic alterations reinforced the distinction between those two tumor types: mutations or fusions of *BRAF* (with another fusion partner than *KIAA1549*) or *RAF1* for DIG/DIA [7], and fusions implicating the RTK genes for IHG [2, 11, 18, 19, 20, 21, 22]. However, and despite numerous molecular analyses, a genetic alteration may be lacking, as was found for one case in our series of DIG/DIA (having a calibrated score of 0.99 for this MC). Despite these differential features, a potential grey zone may exist between these two entities as was revealed by one case from our series presenting histopathological and genetic features of DIG/DIA (with *RAF1* fusion), but classified as IHG by DNA‐methylation profiling (v12.5), and which finally clustered between IHG and DIG/DIA by t‐SNE analysis. This specimen illustrates the utility of integrated diagnoses including radiological, histopathological, genetic, and epigenetic features. As Clarke et al. evidenced, this case highlights that epigenetic boundaries between these two tumor types are not well established and the WHO classification may need to adapt its essential criteria [11]. Additional series are needed to conclude if the genetic dichotomy between IHG and DIG/DIA is confirmed by DNA‐methylation profiling. Because a subset of IHG may present an ependymal differentiation [11, 23], it constitutes a potential diagnostic pitfall for supratentorial ependymomas (*ZFTA* or *YAP1* fusion‐positive), which, according to the results of the current series are not exceptional (representing 13% of the whole cohort) in this age group.

This study also highlights the fact that other differential diagnoses sharing histopathological and genetic features with the two main tumor types encountered in this age group rarely present an infantile age at diagnosis. Firstly, as reported recently, PXA may share genetic features (RTK gene fusions) with IHG [11, 12]. Including the results of the current series, infantile case (<1‐year‐old of age) of PXA may present *ALK*, *ROS1*, *NTRK2*, and *NTRK3* fusions [11, 15, 22], and other cases with *NTRK2* [11, 12], and *ALK* [12] have been described in pediatric age groups. The diagnosis of PXA is difficult by imaging (it bears a close resemblance to IHG), but may be easily made using histological morphology and genetics (the presence of a *CDKN2A* homozygous deletion is found in reported cases [12, 22] and ours, but is absent in the other tumor groups encountered in this age group). Because PXA and IHG may share the same fusions (*PPP1CB::ALK*, *GOPC::ROS1*, and *TPM3::NTRK1*) [11, 12, 15], these results reinforce the diagnostic utility of searching for a homozygous deletion of *CDKN2A* for the diagnosis of PXA. PA may also represent a potential diagnostic pitfall. Indeed, it may present histopathological features of malignancy (mitoses, necrosis, and microvascular proliferation) [24] and MAPK (particularly *BRAF*) or RTK (particularly *NTRK2*) [25] gene fusions. Despite these shared features with IHG or DIG/DIA, a previous study [24] showed that histopathological signs of aggressiveness in PA are insufficient to suggest a diagnosis of HGG, particularly for the pediatric population, based on clinical and DNA‐methylation profiling data. The current results reinforce the idea that the *KIAA1549::BRAF* fusion seems to be highly suggestive for a diagnosis of PA (in case of the absence of 1p deletion). To our knowledge, no hemispheric presentation of an infantile PA (proven by genetic and epigenetic analyses) has been reported, and only supratentorial midline cases have been described. This anatomic distinction may constitute a differential element from IHG and DIG/DIA which are always attached to the dura with a cortical involvement. More recently, a novel MC of glioneuronal tumor (called the “glioneuronal tumor, not otherwise specified, subtype A") harboring RTK (*ALK*, *NTRK1*, *NTRK2*, *NTRK3*, and *MET)* fusions and MAPK (*RAF1*) fusions have been isolated by DNA‐methylation profiling [14, 16]. One of them concerned an infant having an *ALK* fusion [16], which constitutes an additional differential diagnosis for this age group.

Three cases from the current series presented histopathological (diffuse growth pattern, oligodendroglioma‐like morphology, calcifications for two of them, and CD34 immunoreactivity for two of them) and genetic (presence of a *FGFR3::TACC3* fusion in two of them) features reminiscent of a diagnosis of PLNTY [26]. However, two of them presented histopathological signs of aggressiveness (high mitotic count and proliferative index) which are not compatible with the definition of PLNTY in the current WHO classification [1]. Moreover, no infantile case of DNA‐methylation confirmed PLNTY has been described in the literature yet. Their imaging characteristics were strikingly different from other infantile tumors, as well as from known radiological characteristics of PLNTY. Finally, these three cases clustered within the MC of ganglioglioma using t‐SNE analysis as reported in one recent series [27]. Like PA, it seems that infantile forms of ganglioglioma may present histopathological signs of malignancy.

This study underlines the existence of a new type of embryonal tumor, the “CNS embryonal tumor, with PLAG‐family amplification” which is not yet included in the WHO classification. This entity has been defined by a distinct DNA‐methylation profile [28], and is notably different from neuroepithelial tumors, *PLAGL1*‐fused, which present histopathological similarities to ependymomas and harbor unique fusions of the *PLAGL1* gene [29]. The PLAG (pleomorphic adenoma gene) family includes *PLAG1*, *PLAGL1* (PLAG like 1), and *PLAGL2* (PLAG like 2) genes. The name of these genes reminds us that initially, *PLAG1* fusions were implicated in the tumorigenesis of pleomorphic adenoma of the salivary glands [30]. In the CNS, gains and amplifications of these genes have also been reported in gliomas, and it has been evidenced that PLAGL2 promotes renewal of neural stem cells/progenitor cells by inhibiting their differentiation and thus promoting gliomagenesis [31]. Similarly to the two specimens in the current study, the reported cases with *PLAGL2* amplification mainly affected infants and were supratentorial [28, 32]. This case and ours presented an amplification of the *PLAGL2* gene. Radiologically, they appear as a solid and cystic peri‐ or intraventricular mass with an intermediate restriction of the diffusion, high perfusion value, and an intense enhancement following gadolinium injection. Histopathologically, the tumors are well‐circumscribed with a branching capillary network, but without microvascular proliferation, as previously described [28]. The tumor cells are monotonous without a distinct pattern of differentiation. The immunoprofile includes the expression of glial and neuronal markers. Necrosis is inconstant, and the mitotic count and proliferative labeling index are variable among cases. In the current series, the prognosis is varied (one infant died rapidly whereas the other was alive 27 months after the initial diagnosis) and further cases are needed to determine the potential outcome associated with this tumor type.

In conclusion, this cohort highlights that infantile supratentorial tumors encompass several histopathological and molecular types of glial and glioneuronal tumors beyond IHG and DIG/DIA. This heterogeneous group of tumors includes entities presenting histopathological or genetic overlaps with these two main diagnoses. This work underlines the need for a comprehensive analysis (including radiological, histopathological, genetic, and epigenetic data) to obtain an accurate diagnosis. The diagnostic criteria for some entities (PA, ganglioglioma, and PXA), based currently on only histopathological features, seem to be insufficient for this age group. These results reinforce the idea that deciphering the genetic/epigenetic spectrum of infantile tumors is necessary for an adequate risk stratification and therapeutic approach (different relevant genetic targets) in the future.

## AUTHOR CONTRIBUTIONS

Kévin Beccaria, Volodia Dangouloff‐Ros, Nathalie Boddaert, Jacques Grill, Cassandra Mariet, and Franck Bourdeaut compiled the MRI and clinical records. Arnault Tauziède‐Espariat, Alice Métais, Fabrice Chrétien, and Pascale Varlet conducted the neuropathological examinations. Arnault Tauziède‐Espariat, Philipp Sievers, Romain Appay, Raphaël Saffroy, Daniel Pissaloux, and Alexandra Meurgey conducted the molecular studies. Arnault Tauziède‐Espariat, Kévin Beccaria, Volodia Dangouloff‐Ros, Nathalie Boddaert, and Pascale Varlet drafted the manuscript. All authors reviewed the manuscript.

## FUNDING INFORMATION

The authors declare that they have received funding from the Association pour la Recherche en Neurochirurgie Pédiatrique (ARNP).

## CONFLICT OF INTEREST STATEMENT

The authors declare no conflicts of interest.

## ETHICS STATEMENT

This study was approved by the GHU Paris Psychiatry and Neurosciences, Sainte‐Anne Hospital's local ethic committee.

## Supporting information


**Table S1.** Clinical and histomolecular data.

## Data Availability

The data that support the findings of this study are available from the corresponding author upon reasonable request.
